# Research on the Sustainability of Desert Sand-Recycled Concrete Based on the NMR Porosity Structure and Grey Correlation Analysis

**DOI:** 10.3390/ma19122432

**Published:** 2026-06-06

**Authors:** Xinjie Wang, Wenbang Zhu, Yali Cao, Chuikan Li, Ruiming Liu, Enze Hao, Ziyang Cheng, Xiumei Zheng

**Affiliations:** 1 College of Civil Engineering, Kashi University, Kashi 844006, China; 15736743027@163.com (X.W.); wenbangzhu@126.com (W.Z.); 17393129591@163.com (Y.C.); ruiming_liu@163.com (R.L.); 13598804873@163.com (E.H.); 18890939149@163.com (Z.C.); 2 Xinjiang Key Laboratory of Engineering Materials and Structural Safety, Kashi University, Kashi 844006, China; 3 Kashi Zhengxin Construction Engineering Testing Co., Ltd., Kashi 844199, China; 15599917788@163.com

**Keywords:** desert sand, recycled concrete, resistance to chloride ion penetration, grey relational analysis

## Abstract

To investigate the mechanism by which a combination of desert sand (DS) and recycled coarse aggregate (RA) affects the sustainability of recycled concrete, multiple mix proportions with varying replacement ratios were designed in this study. Macroscopic performance tests and microscopic analyses were performed using nuclear magnetic resonance (NMR), scanning electron microscopy (SEM), and X-ray diffraction (XRD), and the results were combined with grey relational analysis to reveal the intrinsic relationships between pore parameters and macroscopic properties. Additionally, technical and economic evaluations were conducted. The results indicate that incorporating either type of aggregate individually has a nonlinear effect on the compressive strength and impermeability of concrete. The optimal compressive strength is achieved when both aggregates are used at 20% replacement, whereas the best impermeability occurs at 10% replacement for each. The proportions of transitional pores and capillary pores, along with T_2_ relaxation parameters, serve as key microstructural indicators for controlling performance. Economically, the use of both aggregates together significantly reduces material costs—reaching a cost savings rate of 3.51% with a recycled aggregate replacement level of 30%. Further substitution of 30% desert sand for river sand under the same replacement ratio can reduce costs by an additional 1.56%. This mix proportion achieves optimal synergy among mechanical performance, cost control, and low-carbon benefits. The findings provide theoretical guidance and practical support for mix design, durability enhancement, and the promotion of such green, low-cost concrete in engineering applications.

## 1. Introduction

Amidst the rapid expansion of the construction industry in recent years, the massive generation of construction waste and the excessive exploitation of sand and gravel resources have posed significant challenges to the ecological environment [1,2,3]. Empirical data indicate that more than 80% or even more than 99% of building material becomes construction waste. Moreover, the global consumption of sand and gravel resources has been increasing annually and is expected to reach 60 billion tons by 2030 [4,5].

Against this backdrop, many scholars have applied recycled aggregates to concrete and reported that the rough surface of recycled aggregates can increase the friction coefficient with cement [6,7,8]. Moreover, the high water absorption rate of recycled aggregates can cause internal curing during the concrete curing process, thereby enhancing the compressive strength of the concrete [9]. However, owing to the high porosity, weak bonding surface of old mortar, low density, and relatively low strength of recycled aggregates [10,11,12], different scholars have drawn opposing conclusions from their related experiments. For instance, Zheng et al. [13] reported that as the proportion of recycled aggregates increased, the basic mechanical properties of recycled concrete decreased significantly. Studies have shown that incorporating an appropriate amount of desert sand can accelerate the early hydration process within cement through particle dispersion, microfilling, and nucleation effects, thereby enhancing the early mechanical properties of recycled concrete [14]. However, few studies have investigated desert sand recycled aggregate concrete (DRC), and the specific influence of desert sand on the mechanical properties and chloride ion penetration resistance of recycled aggregate concrete remains to be elucidated.

Some scholars have studied the impact of different replacement ratios of RA on the performance of concrete [15]. André et al. [16] reported that increasing the RA content reduced the density and workability, while the mechanical properties remained consistent when the RA replacement rate was 20%. Wu, ER et al. [17] reported that the best strength was achieved when the replacement ratio of recycled aggregates was 40%. However, many studies have shown that the incorporation of recycled aggregates usually reduces the chloride ion permeability of concrete and that the chloride ion diffusion coefficient increases with increasing replacement rate [18,19].

Some scholars have researched the performance of concrete with different DS replacement rates [20,21]. Akhtar et al. [22,23] developed sustainable sand using desert sand and recycled fine aggregates that is not only economically feasible but also environmentally beneficial. Hussein M et al. [24] reported that the 28-day compressive strength of concrete was the highest (61.06 MPa) when the replacement rate of desert sand was 50%. Hamada, HM et al. [24] reported that the compressive strength was the highest when the replacement of desert sand was 50%. Ji et al. [25] studied the mechanical properties of recycled rubber desert sand concrete and reported that the compressive strength was the highest when the replacement rate of desert sand was 20%. Liu et al. [26] investigated the mechanical properties and pore structure of desert sand concrete and established a mechanical property degradation model. Kazmi [27] and Xu et al. [28] studied the durability of desert sand concrete in a sulfate-rich environment, but few studies have investigated the influence of the DRC on the chloride ion permeability [29]. Liang et al. [30] investigated the influence of recycled coarse aggregate characteristics on the elastic modulus of recycled concrete through experimental and numerical simulations, explicitly considering the material weakening effect—namely, the reduced mechanical performance of old mortar due to microcracks and higher porosity. The results indicated that the elastic modulus of the old mortar is the dominant factor affecting the overall elastic modulus. Although these studies have provided important foundations for evaluating the performance of recycled aggregate concrete, research on blended systems involving multiple types of recycled aggregates remains very limited.

Chen et al. [31] utilized nuclear magnetic resonance (NMR) technology to quantitatively capture the changes in porosity and pore size distribution within concrete and revealed the mechanism through which the freeze–thaw durability of concrete is impacted at the microstructural level. Wang et al. [32] studied the pore structure deterioration of RPC using NMR and scanning electron microscopy (SEM). Therefore, to explore the mechanism through which the durability of DRC is affected, this study quantitatively characterized its multiscale pore structure using NMR technology and evaluated its chloride ion penetration resistance through electrical flux tests. On this basis, the grey relational analysis method was applied to systematically study the degree of correlation between key NMR pore parameters such as porosity and pore size distribution and the chloride ion migration performance [33,34]. Through the analysis of technical and economic sustainability, a quantitative assessment of the comprehensive sustainability of DRC is achieved. This study establishes an analytical framework that combines NMR microscopic characterization with grey system theory, clarifying the key pore characteristics in DRC that influence its mechanical properties and chloride ion transport. This provides a theoretical basis and microscopic evaluation method for the durability design and performance optimization of DRC. Multi-index comprehensive performance evaluation offers an ideal basis for selecting materials that balance performance requirements and budget constraints in practical engineering.

## 2. Experimental Materials and Methods

### 2.1. Raw Materials

**Figure 1:**
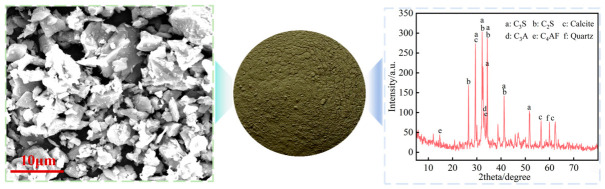
SEM images and XRD patterns of OPC [35].

**Figure 2:**
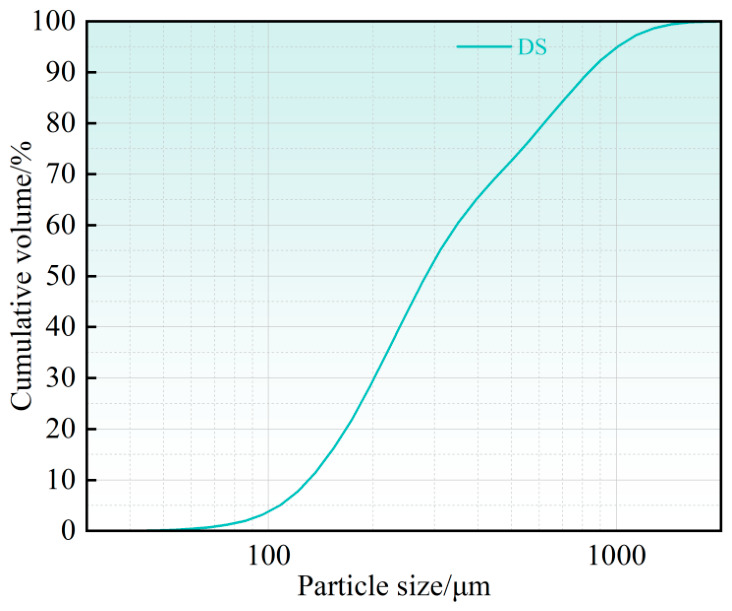
DS Particle Size Distribution Diagram.

**Figure 3:**
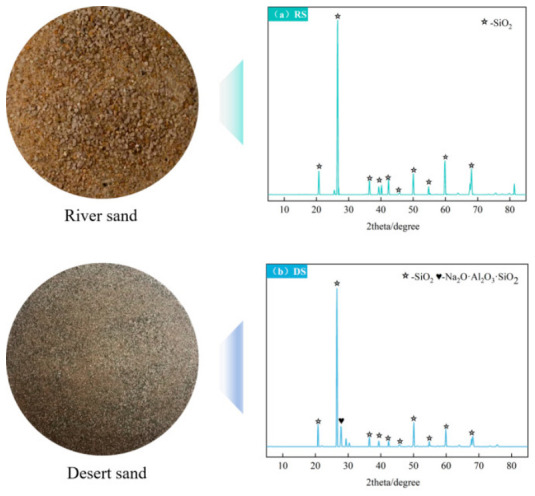
XRD patterns of the RS and DS.

**Figure 4:**
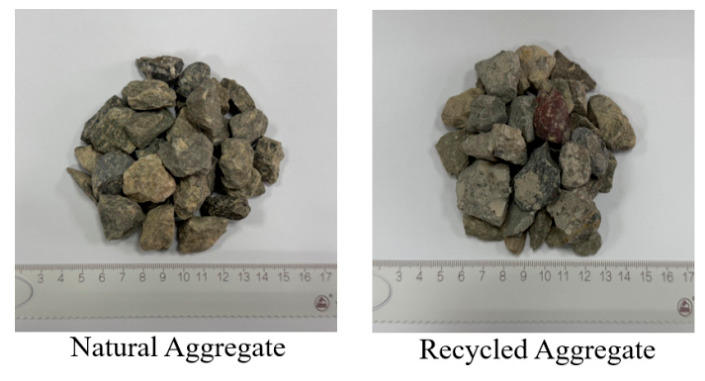
Coarse aggregate.

In this study, a water-to-binder ratio of 0.40 was selected, with Portland cement as the binder. The cement used in the experiments was 42.5 grade ordinary Portland cement (OPC) produced by Kashi Tianshan Cement Co., Ltd. (Kashi, China). The superplasticizer (SP) selected was a high-efficiency water-reducing agent with early strength and delayed setting properties and a water reduction rate greater than 25% produced by Xinjiang Shengxinghe Chemical Co., Ltd. (Urumqi, China). The mixing water was local tap water from the Kashgar region. Since the experiments in this study used cement raw materials of the same manufacturer and specification, the XRD, XRF, and SEM micro-characterization data for the cement were directly adopted from the previous research results of the research group, without repeating the experimental tests. The chemical composition of the cement was analysed using X-ray fluorescence spectrometry (XRF), and the results are presented in Table 1. The microstructure and phase composition of the cement were characterized by scanning electron microscopy (SEM) and X-ray diffraction (XRD), respectively, and the results are shown in Figure 1 [35]. The cement particles were in the shape of irregular blocks, and the main mineral components were tricalcium silicate and dicalcium silicate; however, the internal porosity of the matrix was relatively high and unevenly distributed. The fine aggregates used in the experiments included natural river sand (RS) and desert sand (DS) collected from the surrounding areas of Kashi. The particle size distribution of the DS is shown in Figure 2. The phase composition of the two fine aggregates was analysed by XRD, and the results are shown in Figure 3. From the perspective of chemical and mineral composition, the main component of river sand is SiO_2_; the composition of desert sand is relatively complex, and in addition to the main phase SiO_2_, it also contains a small amount of sodium feldspar [(Na,Ca)Al(Si,Al)_3_O_8_] with potential alkali reactivity. Recycled coarse aggregate (RA) was obtained by crushing and screening the demolition waste with a jaw crusher. Its basic performance indicators and microstructure are shown in Table 2 and Figure 4, respectively.

### 2.2. Mix Proportion Design

**Figure 5:**
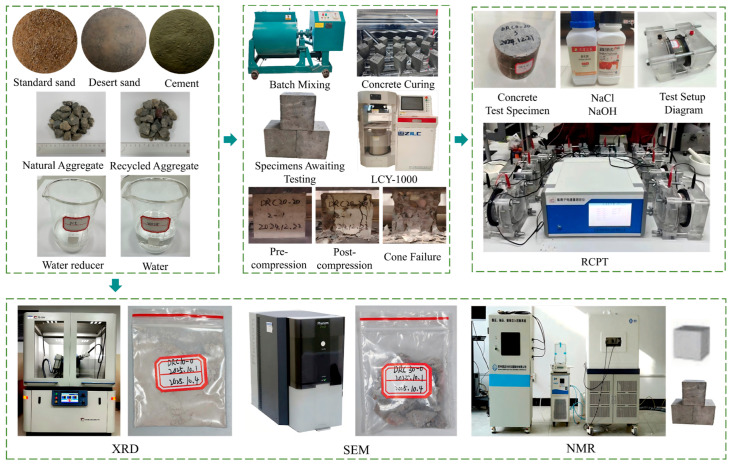
Preparation of DRC samples and the testing process.

In this study, a concrete mix proportion design in accordance with the Chinese standard JGJ55-2019 was used [36]. The substitution rates of DS (0%, 10%, 20%, 30%) and RA (0%, 10%, 20%, 30%) were taken as the experimental variables, and a total of 10 mix proportions were designed. The specific mix proportion parameters are shown in Table 3. The DS substitution rate is the mass ratio of DS to fine aggregate, and the RA substitution rate is the mass ratio of RA to coarse aggregate. After each raw material was weighed according to the designed mix proportion, the aggregates and cementitious materials were first dry mixed in the mixer for 30 s, and then the mixing water and water reducing agent were added for wet mixing for 120 s to ensure the uniformity of the mixture. After the mixing was completed, the test moulds were filled with the concrete mixture and a vibration table was used for compaction. A total of 10 mix proportions were designed for this experiment, with three replicate tests for each proportion. The specimen preparation details are as follows: for each mixture, three chloride ion penetration samples (φ 100 mm × 50 mm), nine compressive strength samples (100 mm × 100 mm × 100 mm) divided into three curing ages (three specimens per age), and three NMR samples (40 mm × 40 mm × 40 mm) were prepared, totalling 150 samples. After demoulding, the samples were covered with film, left to stand at room temperature for 24 h, and then moved to a standard curing room with a temperature of 20 ± 2 °C and a relative humidity of more than 95% for curing. The process of sample preparation and curing is shown in Figure 5.

### 2.3. Test Methods

#### 2.3.1. Mechanical Tests

In accordance with the cube concrete compressive strength test method stipulated in the Chinese national standard GB/T50081-2019 [37], a microcomputer-controlled electrohydraulic servo pressure testing machine (LCY-1000, Zhejiang Lice Instrument & Equipment Co., Ltd., Hangzhou, China) was used to conduct compressive strength tests on 100 mm × 100 mm × 100 mm cubic samples of DRC at 3 d, 7 d, and 28 d of age. The loading rate was controlled at 0.5 MPa/s.

#### 2.3.2. Electrical Flux Test

In accordance with the Chinese national standard GB/T 50082-2024 [37,38], electrical flux tests were conducted on 100 mm × 50 mm cylindrical DRC samples. First, the sample surfaces were ground smooth and placed in a vacuum environment with a vacuum degree of 133 Pa for 3 h. While the vacuum degree remained unchanged, deionized water was injected into the vacuum environment, and then the samples were transferred to a normal pressure environment for soaking for 18 ± 2 h. After soaking, the samples were retrieved, and any surface water was removed by wiping. The sides of the samples were sealed with epoxy resin to prevent water leakage during the test. The electrolyte configuration for the test was as follows: the anode chamber was filled with a 0.3 mol/L NaOH solution, and 3% NaCl solution by mass was injected into the cathode chamber. During the test, a 60 V DC stabilized power supply was applied. Under the action of the electric field, negative ions inside the sample migrated towards the positive electrode and passed through the sample. During the test, current data were recorded every 30 min for a continuous 6 h to obtain complete electrical flux test data.

#### 2.3.3. Microscopic Performance Tests

(1) NMR test

DRC cubic samples (40 mm × 40 mm × 40 mm) that had been cured for 28 d via standard means were selected. They were pretreated using the same vacuum saturation regime used in the electrical flux test. After the treatment, the samples were removed, and their surfaces were dried. Afterwards, the samples were quickly placed in the NMR equipment probe. By collecting the hydrogen nuclear relaxation signals inside the samples, the microscopic characteristic parameters such as the internal pore structure and pore size distribution of the concrete can be inversely analysed.

(2) SEM test

A Phenom Pro X scanning electron microscope (Thermo Fisher Scientific, Eindhoven, The Netherlands) was used to observe the microscopic morphology of the DRC samples. Approximately 1 cm^3^ of the sample was cut from the centre of the sample after the compressive test, immersed in anhydrous ethanol for 24 h to terminate hydration, and vacuum dried at 50 °C until a constant weight was achieved. To prevent the accumulation of electrons on the sample surface from affecting the imaging, an ion-sputtered gold coating was applied to the sample.

(3) XRD test

Samples were taken from the centre of the DRC samples after the compressive test. The same hydration termination and drying treatment method that was used for the SEM samples was employed. The samples were ground into powder and passed through an 80 μm sieve. The test parameters included a tube voltage of 30 kV, a tube current of 20 mA, a scanning range of 5–85°, and a scanning rate of 0.1°/s.

## 3. Analysis of the Test Results

### 3.1. Mechanical Properties of DRC

**Figure 6:**
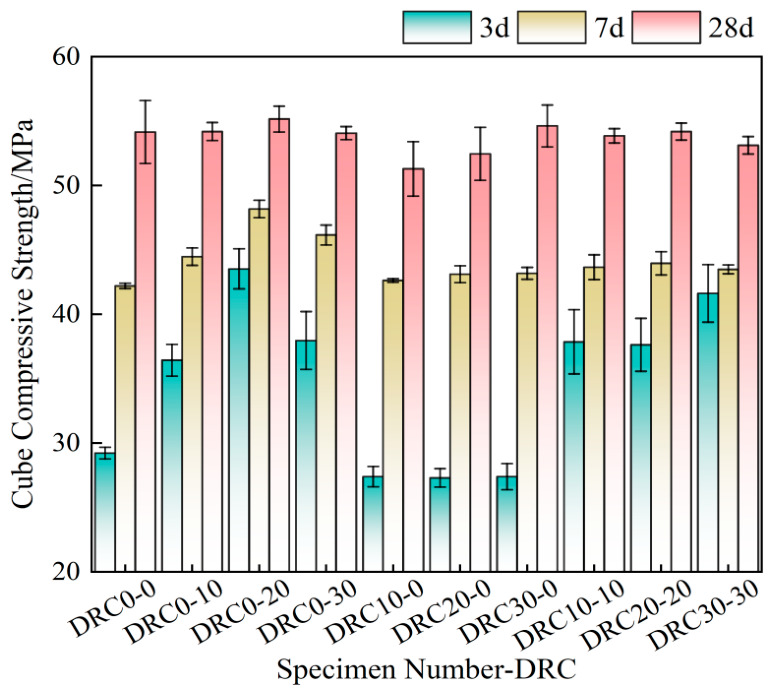
Compressive strength test results.

The evolution of the compressive strength of DRC cubes with different RA and DS replacement rates with increasing curing age is shown in Figure 6. When RA is used as a single additive, the concrete develops distinct longitudinal through-cracks after compressive failure, accompanied by surface mortar spalling, which is a typical splitting failure mode; the compressive strength of the DRC continues to increase with increasing curing age, and the effect of the RA replacement rate on the early strength at 3 d and 7 d is relatively limited. At 28 d, the compressive strength first decreases but then increases with increasing RA replacement rate, and the sample with a 30% RA replacement rate performs the best, with its strength slightly higher than that of the control group. This is because the hydration process gradually compensates for early defects on the surface of the old mortar of the RA as it progresses, and the rough morphology and internal curing effect optimize the performance of the interfacial transition zone. When DS is added alone, the sample exhibits fine and dense crack development, with good overall structural integrity and certain ductile failure characteristics, and the compressive strength of the DRC at each age first increases but then decreases with increasing DS replacement rate. At 28 d of age, the sample with a 20% DS replacement rate has the best performance, which is 1.85% greater than that of the reference group. Low-dose DS can have a microaggregate filling effect that optimizes the gradation and increases the density, whereas high-dose DS will not result in ideal gradation and will increase the porosity, ultimately weakening the load transfer efficiency [39].

When RA and DS were compounded and added, the fracture surfaces of the samples with a high proportion of both additives were rough, and the debonding phenomenon between the aggregates and the cement paste matrix at the interface was more pronounced. The compressive strength of the DRC at the early ages of 3 d and 7 d was higher than that of the control group, whereas at the age of 28 d, only the compressive strength of the sample with a 20% replacement rate was slightly higher than that of the control group. During the early hydration stage of the DRC (3 d and 7 d), because of its high water absorption, the RA can gradually release water to promote cement hydration, and the fine DS particles fill the micropores, increasing the density of the microstructure; in the later stage, the inherent defects of the RA and DS gradually dominate the strength development, and the compressive strength at 28 d tends to decrease again with increasing replacement rate: the negative effect is dominant at low dosages, and the microaggregate filling effect of the DS reaches a peak at a 20% replacement rate, which can effectively offset the defects, whereas at a 30% replacement rate, the defect interface forms a connected network [40], and the negative effect of excessive DS is superimposed, resulting in a significant decrease in strength. In DRC20-20, the replacement rates of DS and RA are both 20%, at which point the compressive strength of the DRC reaches a peak of 54.20 MPa, which is higher than that of the control group, demonstrating the best mechanical performance.

### 3.2. Chloride Ion Permeability Resistance Performance

**Figure 7:**
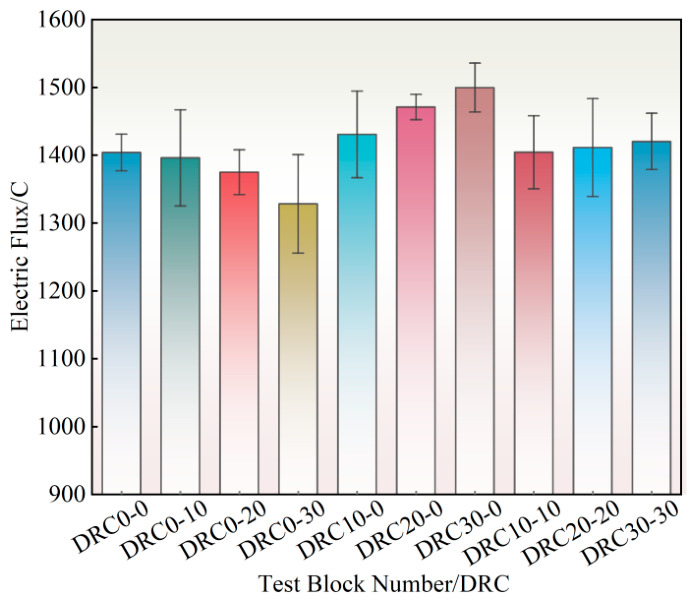
Charge passed.

The electrical flux method (ASTM C1202-12 [41]) used in this study is an accelerated test that integrates diffusion, electromigration, and ionic conductivity of the pore solution, enabling a relative evaluation of chloride ion permeability. Therefore, the chloride ion permeability of the concrete was assessed according to the ASTM C1202-12 [37,42] standard, with the corresponding classification criteria shown in Table 4. According to this standard, the lower the 6 h electrical charge (QS) is, the lower the internal chloride ion permeability of the concrete, and the better its resistance to chloride ion penetration. The 6 h electrical charge test results of the DRC samples with different DS and RA replacement rates are shown in Figure 7. As shown in Figure 7, the QS values of all DRC samples are within the range of 1000 ≤ QS < 2000, which corresponds to the “low” permeability grade. This finding indicates that the DRC matrix remains highly dense after the incorporation of RA and DS; thus, the invasion of harmful media such as chloride ions and water is effectively avoided. Moreover, different replacement rates of RA and DS significantly affect the chloride ion penetration resistance of the DRC. The variation law of the 6 h chloride ion QS values of the DRC under different DS and RA replacement rates is shown in Figure 7. The QS values of all the samples are within the range of 1000 to 2000 °C, which corresponds to the “low” permeability grade, indicating that the overall density of the DRC matrix is relatively high and can effective resist the invasion of harmful media such as chloride ions. Furthermore, regarding the chloride ion transport mechanism, the migration of chloride ions in the DRC is influenced primarily by a combination of factors, including the ionic concentration of the pore solution, pore connectivity, and tortuosity, as well as physical and chemical adsorption of chloride ions onto the pore walls. An in-depth analysis of these aspects has not yet been conducted, but will be addressed through targeted microscopic mechanism research in future work.

**Figure 8:**
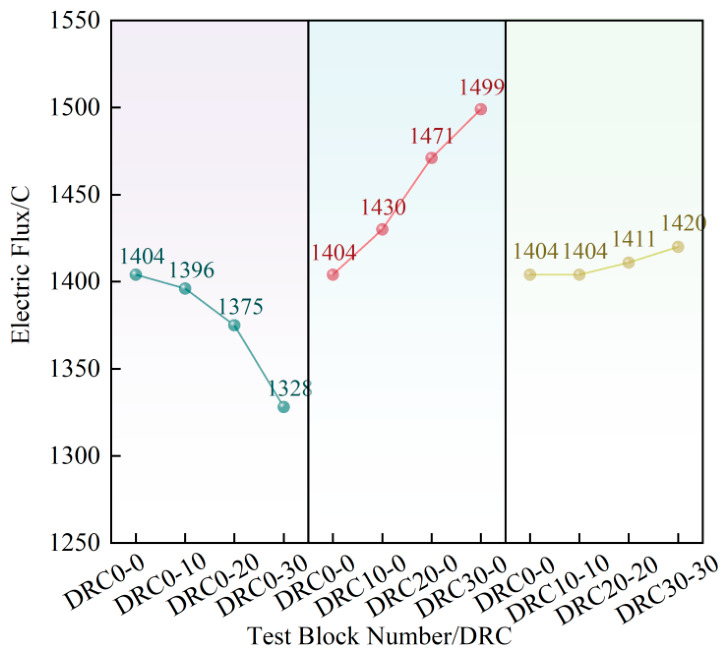
Charge passed at different replacement ratios.

As shown in Figure 8, when DS is added alone, the QS value of the DRC tends to continuously decrease with increasing substitution rate. When the substitution rate of DS is 30%, the QS value decreases to 1328 C, which is a decrease of 5.41% compared with that of the DRC0-0 control group. This is because the desert sand particles are extremely fine and can act as microaggregates to fill the voids in the river sand skeleton, the interface between the river sand and cement paste, and the tiny pores between the cement particles, thereby optimizing the internal pore structure of the concrete. As the substitution rate increases to 30%, the filling effect is continuously enhanced, promoting the transformation of large pores to small pores, significantly reducing pore connectivity, and blocking the channels for chloride ion migration, thus resulting in a decrease in the QS value. When only RA is added, the QS value of DRC tends to gradually increase with increasing substitution rate. When the substitution rate of RA is 10%, the electric flux is 1430 C, an increase of 1.85% compared with that of the control group; when the substitution rate increases to 30%, the QS value increases to 1499 C, an increase of 6.78% compared with that of the control group. This finding indicates that at low dosages, the microfilling effect and internal curing effect of RA are dominant, which can improve the interface structure to a certain extent; however, as the substitution rate increases, the inherent pores of the RA, the weak zones of the old mortar, and the defects at the interface between new and old mortar gradually accumulate, and their negative effects outweigh the early positive effects, leading to an increase in internal permeation channels and a significant increase in the QS value.

When DS and RA are both added, the QS value of the DRC tends to slowly increase as the substitution rate increases. When the substitution rates of DS and RA are both 10%, the QS value of the sample is the same as that of the control group. At this stage, the microaggregate filling effect of DS and the interface defects introduced by RA form a dynamic balance, and the particle gradation is close to the ideal continuous distribution. RA, as a coarse aggregate, combined with river sand, DS, and cement particles can be used to construct a dense skeleton, effectively reducing the total porosity and connectivity of the system. As the substitution rate further increases, the high porosity and weak interface defects of the old mortar on the surface of the RA gradually become dominant, and their negative effects far outweigh the pore refinement effect of DS, resulting in an increase in internal permeation channels, and the electric flux continuously increases with increasing substitution rate.

### 3.3. Research on Microscopic Performance

#### 3.3.1. NMR Analysis

**Figure 9:**
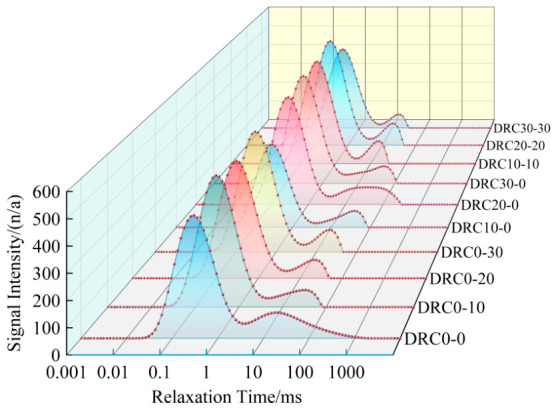
Transverse relaxation-time distribution of DRC.

Before the NMR and XRD tests were conducted, triplicate parallel sampling measurements were performed for each mixed sample. The test results showed good overall consistency; therefore, a single representative test result with typical characteristics was selected as the final data for presentation in this paper. The NMR T_2_ relaxation spectra distributions of the DRC under different DS and RA replacement rates are shown in Figure 9. As shown in the figure, all the samples have two characteristic signal peaks in the T_2_ spectrum, which correspond to the pore structures of different sizes inside the concrete. With increasing DS and RA replacement rates, the total area of the T_2_ spectrum peaks generally tends to decrease, and the peak area of each group does not exceed that of the control group, indicating that the total pore volume inside the samples without the addition of DS and RA is relatively high and that the addition of both DS and RA has a significant regulatory effect on the pore characteristics of the DRC. In the relaxation time range of 300 to 1200 ms, which corresponds to large pores, only the control group shows a relatively high signal peak intensity, and the sample with a 20% RA replacement rate retains a weak signal, while essentially no large pore signals are observed for the other groups. These results indicate that, except for the sample with a 20% RA replacement rate, the other combinations of DS and RA dosages effectively fill the large pores inside the DRC, and when the RA replacement rate is 20%, the particle size distribution and the matrix voids are well matched, and the proportion of large pores does not decrease significantly. In the relaxation time range of 0.7 to 2.3 ms, which corresponds to that of the gel pores, the sample with DS and RA replacement rates of 20% each has the highest signal peak intensity; moreover, in the large pore range of 400 to 1200 ms, the signal peak intensity of the samples is 0. These findings indicate that when both the DS and RA replacement rates are 20%, the proportion of small pores can significantly increase, and the number of large pores can decrease, resulting in a refined optimization of the pore size distribution inside the concrete and further revealing the microscopic mechanism by which DS and RA synergistically improve the mechanical properties and chloride ion penetration resistance of the DRC from the NMR perspective [43].

**Figure 10:**
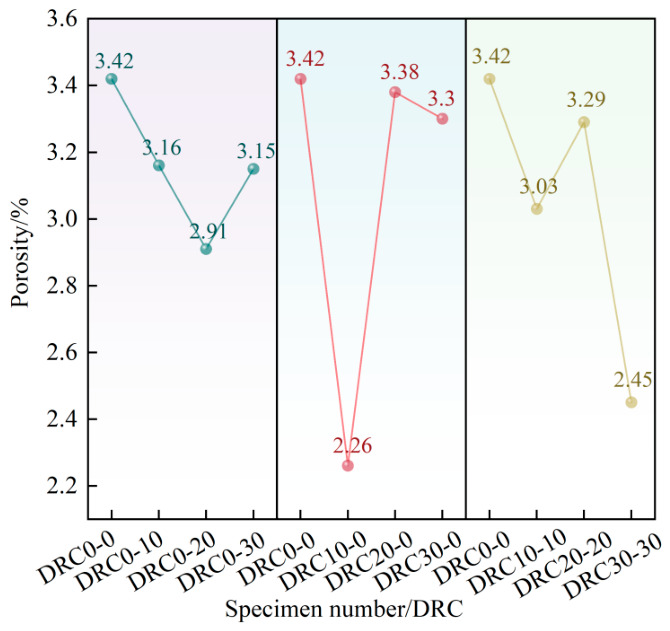
Porosity of DRC.

The variation trend in the porosity of DRC samples with different mix ratios is shown in Figure 10. As shown in Figure 10, when DS is added alone, from the control group DRC0-0 to DRC0-30, the porosity tends to first decrease but then increase: the porosity decreases from 3.42% for the control group to 2.91% for DRC0-20 and then increases to 3.15% for DRC0-30. Among them, DRC0-20 has the lowest porosity, indicating that its internal structure is relatively dense and that the proportion of harmful large pores is relatively small, whereas the porosity of the control group DRC0-0 is the highest, indicating that the pore development of the matrix is more complete when DS is not added. When RA is added alone, from the control group DRC0-0 to DRC30-0, the porosity first sharply decreases but then increases with increasing RA replacement rate: it rapidly decreases from 3.42% to 2.26% for DRC10-0 and then increases to reach a value close to that of the control group. The porosity of DRC10-0 is significantly lower than that of the other groups, which can be attributed to the following: at a low replacement rate, the particle size distribution of RA can effectively fill the large pores inside the matrix, and the old mortar adhering to its surface forms a good bond with the new cementitious material, making the overall structure denser and thus reducing the porosity [16]; at a high replacement rate, the internal pores of RA increase, and the defects in the interface transition zone between RA and the new matrix intensify, resulting in an increase in the overall porosity [44]. This microscopic rule verifies the superior 28-day compressive strength of the DRC10-0 group. When DS and RA are added together, from the control group DRC0-0 to DRC30-30, the porosity first decreases to 3.03%, then slightly increases to 3.29%, and then sharply decreases to 2.45%, among which DRC30-30 has the lowest porosity. The underlying mechanism is as follows: the filling effect of DS microaggregates and the skeleton support effect of RA create a synergistic effect; DS can directly fill the large pores between RA particles, and at the same time, the formation of new large pores is limited by the mixed system, and, to a certain extent, it repairs the pore defects and optimizes the pore structure [14]. The experimental results indicate that total porosity is an important microstructural parameter for determining the chloride ion penetration characteristics of concrete but cannot fully characterize the impermeability and durability of the material. Pore connectivity and pore tortuosity also significantly influence chloride ion transport behaviour: connected pores create continuous pathways for ion migration, accelerating the penetration of aggressive media, whereas highly tortuous pores effectively lengthen the actual diffusion path of chloride ions, thereby suppressing ion transmission rates. Analysis of our experimental data reveals that some samples with low porosity do not exhibit optimal resistance to chloride penetration, further confirming the critical role of pore structure morphology in controlling permeability. In summary, the resistance of the concrete to chloride ion penetration is jointly controlled by the total porosity, pore connectivity, and pore tortuosity. By optimizing the porosity of the matrix and further improving the spatial distribution characteristics of the internal pore structure, the overall durability of the material can be effectively enhanced.

**Figure 11:**
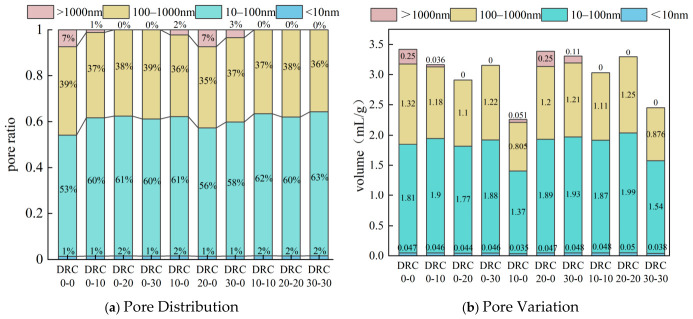
Pore Distribution and Variation.

High porosity significantly reduces the density of concrete and increases the interconnection of internal pores, thereby lowering the compressive strength and resistance to chloride ion penetration and accelerating the ingress of external aggressive agents. In this study, owing to the addition of recycled coarse aggregate (RA) and desert sand (DS), the porosity of all the mix proportions was lower than that of ordinary concrete. A comparison of the total porosity data of the samples in Figure 11 reveals that the highest total porosity occurred when the RA replacement rate was 20% without DS (DRC20-0 group), which was the most unfavourable pore structure condition. This group also exhibited poor performance in both compressive strength and impermeability tests, further confirming the negative effects of high porosity on concrete structures. Additionally, the DRC30-30 group had the lowest total porosity among all the mixtures except for DRC10-0, yet its compressive strength and impermeability were inferior to those of the DRC20-20 group and some mixtures with higher porosities. This anomalous behaviour may be attributed not only to the introduction of numerous original microcracks and weak interfacial zones by the high replacement rate of RA, which serve as preferential pathways for chloride ion diffusion, but also possibly to the formation of continuous pore networks that enhance connectivity, preventing a corresponding improvement in impermeability despite reduced porosity. In summary, the interaction of the DS and RA replacement rates has a significant regulatory effect on the evolution of the internal pore structure of the samples. To further elucidate the formation mechanism of the porosity fluctuation rule, a detailed analysis of the pore size distribution characteristics will be conducted in a subsequent study.

**Figure 12:**
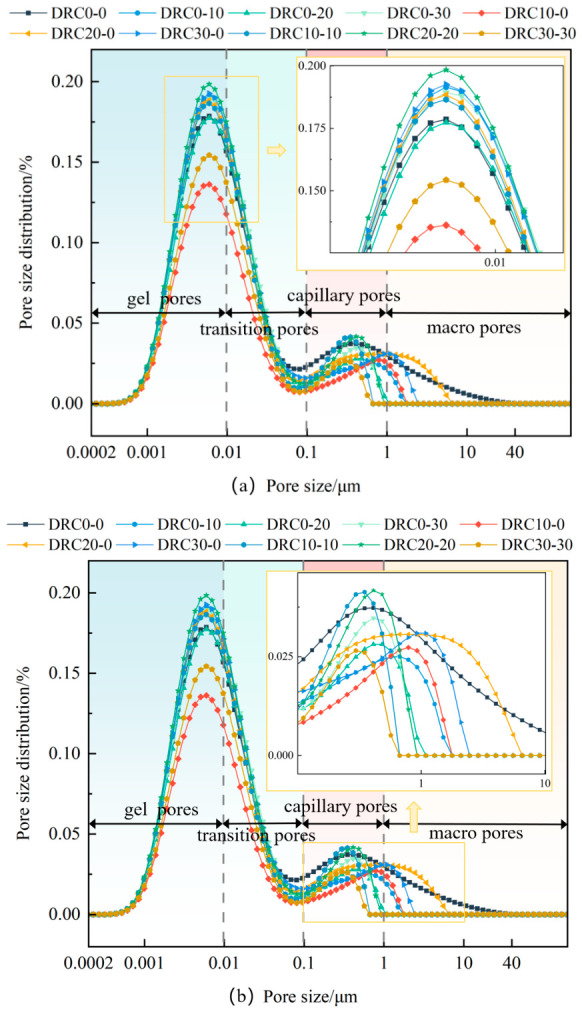
Pore size distribution at 28 days.(**a**) Pore size/um, (**b**) Pore size/um.

On the basis of pore size, the internal pores of concrete can be classified into four categories [45]: pores with diameters less than 10 nm are gel pores, those with diameters between 10 and 100 nm are transition pores, those with diameters between 100 and 1000 nm are capillary pores, and those with diameters larger than 1000 nm are large pores. The pore size distribution characteristics of DRC under different DS and RA replacement rates are shown in Figure 12. When only DS is added, the proportion of small pores (gel pores and transition pores) in each sample varies slightly, while the proportion of large pores (capillary pores and large pores) is significantly lower than that of the control group DRC0-0. As the DS replacement rate increases, the peak values of the large pores in DRC0-10, DRC0-20, and DRC0-30 decrease by 32.80%, 24.46%, and 6.72%, respectively, compared with that in the reference group. This finding indicates that the large pores in the samples can be effectively controlled by adding an appropriate amount of DS, but excessive DS weakens the pore control effect. When only RA is added, the proportion of small pores changes significantly with increasing replacement rate: as the RA replacement rate increases, the number of small pores first decreases significantly but then rebounds. The peak value of small pores in DRC10-0 is 23.74% lower than that in the reference group, while those in DRC20-0 and DRC30-0 are 5.49% and 7.84% higher than that in the control group, respectively. This suggests that a low dosage of RA has a more significant effect on controlling small pores, whereas excessive addition has the opposite effect. When both DS and RA are added, the peak value of small pores first increases but then decreases with increasing replacement rate. The peak value of small pores in DRC30-30 is 13.61% lower than that of the control group, and the decrease in the peak value of large pores in this group is the most significant among all the groups. In conclusion, the pore size distribution characteristics of concrete directly determine its structural compactness: the higher the proportion of small pores is, the better the macroscopic mechanical properties and the resistance to chloride ion penetration of the concrete; an excessive proportion of large pores will form weak channels, leading to performance deterioration [32,46].

#### 3.3.2. SEM Analysis

**Figure 13:**
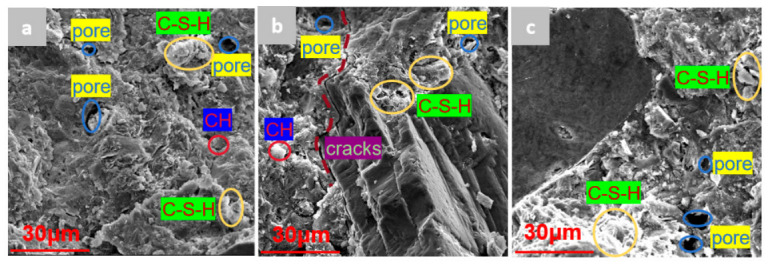
Microstructures with RA substitution: (**a**) DRC0-0; (**b**) DRC10-0; (**c**) DRC30-0.

To reveal the intrinsic connection between the mechanical properties and the resistance to chloride ion penetration of DRC and its microstructure, SEM observations were conducted. The microstructure of the DRC with the addition of only RA is shown in Figure 13. For the control group DRC0-0, the hydration products are mainly C-S-H gel and Ca(OH)_2_, which are uniformly distributed and have dense structures. The natural aggregate and the paste interface is well bonded. When the RA content is 10%, the hydration products are dispersed, and the porosity is significantly reduced, but a few microcracks are present, resulting in a decrease in the compressive strength. When the RA content is 30%, the hydration products aggregate on the surface of the RA, and the porosity slightly decreases. At a high RA content of 30%, the increase in RAs leads to the formation of a connected network of pores on the surface of the old mortar and weak interfaces, accelerating the migration of chloride ions. Therefore, with increasing RA content, the QS value continuously increases, and DRC30-0 has the poorest resistance to chloride ion penetration [47,48].

**Figure 14:**
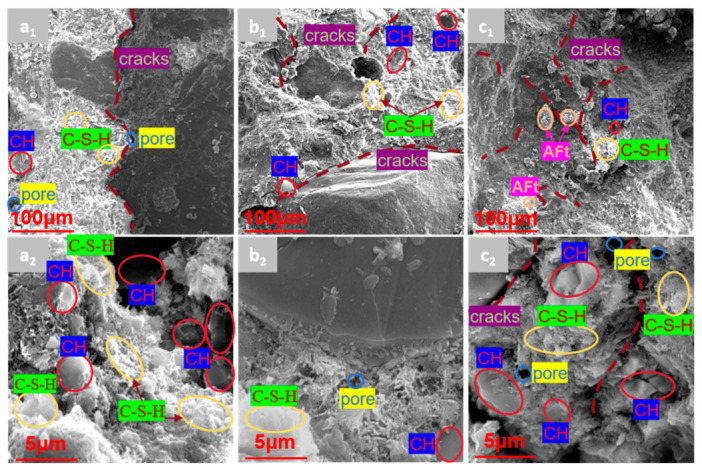
Microstructures with DS substitution: (**a_1_**,**a_2_**) DRC0-10; (**b_1_**,**b_2_**) DRC0-20; (**c_1_**,**c_2_**) DRC0-30.

SEM micrographs of the DRC at different DS substitution rates are shown in Figure 14. When the DS substitution rate is 10% or 20%, the main hydration products of the samples are C-S-H gel and Ca(OH)_2_ crystals, which are evenly distributed. The porosity gradually decreases with increasing DS dosage. The microaggregate filling effect of DS effectively optimizes the particle gap and interfacial transition zone, increasing the density of the matrix more and gradually increasing the compressive strength [14,35]. When the DS substitution rate reaches 30%, a large amount of ettringite crystals (AFt) are generated in the system. Although the porosity further decreases, the number of microcracks significantly increases, resulting in a decrease in compressive strength. The electrical flux depends mainly on the pore connectivity. With increasing DS substitution rate, the microcracks become dispersed, and forming a continuous channel becomes difficult. In addition, the pore refinement and blocking effects of DS continuously increase the resistance of the concrete to chloride ion penetration, and the QS value of the electrical flux continuously decreases.

**Figure 15:**
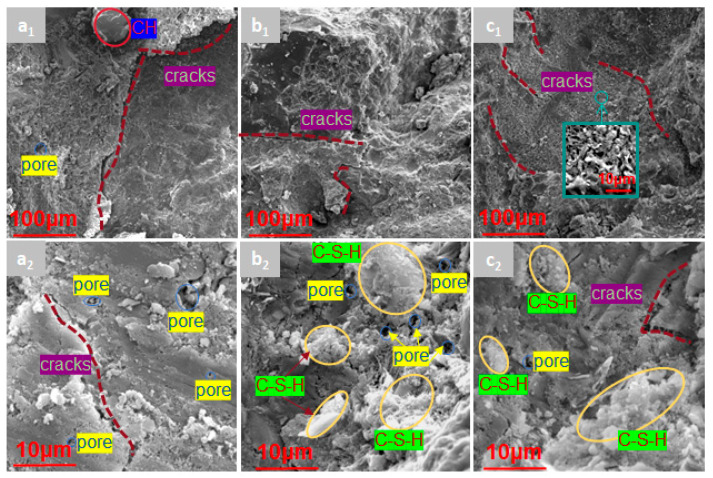
Microstructures with recycled aggregate and desert sand substitution: (**a_1_**,**a_2_**) DRC10-10; (**b_1_**,**b_2_**) DRC20-20; (**c_1_**,**c_2_**) DRC30-30.

The SEM microstructures of the DRC when DS and RA are codoped in the same proportion are shown in Figure 15. When the substitution rate is 10% for both, small amounts of C-S-H gel and Ca(OH)_2_ crystals are generated in the sample, and there are obvious microcracks, with the compressive strength slightly lower than that of the control group. When the substitution rate is 20%, the matrix structure is dense, a large amount of C-S-H gel is generated, and the compressive strength is significantly improved. When the substitution rate is 30%, although C-S-H gel is the main product, many microcracks develop, and the compressive strength is significantly reduced. At low dosages, more pores are present in the samples, and although the porosity is reduced at high dosages, the number of microcracks increases significantly. Therefore, at a substitution rate of 10%, the chloride ion permeability is the lowest, and the impermeability performance is the best, whereas at a substitution rate of 30%, the QS value is the highest, and the impermeability performance is the worst.

#### 3.3.3. XRD Analysis

**Figure 16:**
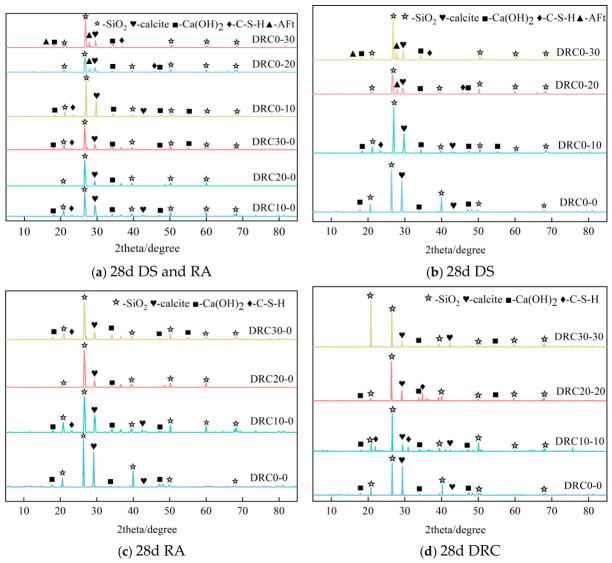
XRD pattern of DRC.

XRD analysis was conducted on DRC samples with different DS and RA dosages, and the results are shown in Figure 16. The main hydration products of the DRC include SiO_2_, C-S-H, Ca(OH)_2_, calcite (CaCO_3_), and AFt. The intensities of the diffraction peaks of each phase differ slightly with differences in the DS and RA dosages, which is related to the pozzolanic effect of the two [49,50]. Without the addition of DS and RA, the main diffraction peaks of the sample are SiO_2_, Ca(OH)_2_, and calcite. When only DS is added, in addition to the above phases, the diffraction peaks of C-S-H and AFt are significantly enhanced. This is because the sulfate components in DS can increase the concentration of sulfate ions in the slurry, providing raw materials for the formation of ettringite, thereby promoting the extensive formation of AFt and C-S-H. When only RA is added, the diffraction peak of C-S-H in the sample is significantly enhanced. The unhydrated cement particles in the old cement slurry coating on the surface of the RA rehydrate in the fresh concrete upon contact with water, generating additional C-S-H gel, which optimizes the pore structure, increases the proportion of transition pores, and reduces the volume of large pores and the total porosity. Moreover, the old cement paste adhering to the surface of the recycled aggregate (RA) contains small amounts of sulfate and various other ions. Changes in the concentrations of these ions can indirectly affect the hydration process and internal microstructure of recycled aggregate concrete (DRC), thereby influencing the mechanical properties of the concrete accordingly. These results are consistent with those of the nuclear magnetic resonance analysis, verifying from the phase perspective the mechanism by which RA improves the mechanical and durability properties of concrete.

## 4. Technical and Economic Sustainability Analysis

To explore the feasibility of the safe and efficient application of DRC in engineering, this study established a comprehensive evaluation system that combines technology and economy. On the technical level, on the basis of grey relational analysis, the correlations between the compressive strength and chloride ion permeability of the concrete and their key influencing factors were identified. On the economic level, material cost and carbon benefits were taken as the core indicators. Ultimately, through the construction of a comprehensive technical and economic index, a quantitative assessment of the overall sustainability of DRC was achieved.

### 4.1. Technical Analysis

Grey relational analysis is defined as a multivariate statistical technique specifically designed to address complex systems characterized by small sample sizes and incomplete information and is used to analyse the degree of correlation among various factors in a system [8]. This study aims to investigate the application of grey system theory in evaluating the mechanical properties and chloride ion penetration resistance of concrete and to assess its effectiveness [51]. In this study, MATLAB R2024b is used to calculate the grey relational degree. The main sequence is represented as follows: *Y* = {*Y_(k)_* | *k* = 1, 2, …, *n*}; the subsequences are represented as follows: *X_i_* = {*X_i_*(*k*) | *k* = 1, 2; *i* = 1, 2, …, *m*}. In this study, the compressive strength *Y*_1_ and the chloride ion penetration resistance parameter *Y*_2_ of the DRC samples are regarded as the main sequences. The subsequences of *Y*_1_ consist of the total porosity *X*_1_, transition pores *X*_2_, capillary pores *X*_3_, and T_2_ spectrum area *X*_4_ of the DRC0-0, DRC0-20, DRC30-0, and DRC20-20 groups of specimens at 28 d of age [52,53]. The subsequences of *Y*_2_ consist of the total porosity *X*_1_, transition pores *X*_2_, capillary pores *X*_3_, and T_2_ spectrum area *X*_4_ of the DRC0-0, DRC0-30, DRC10-10, and DRC20-20 groups of specimens at 28 d of age.

Dimensionless transformation of the data is shown in Equation (1):

(1) $$x_{i}^{\prime }\left(k\right)=\frac{x_{i}\left(k\right)}{x_{i}\left(1\right)},k=1,2\ldots \ldots ,n;i=1,2,\ldots \ldots ,m$$

where *x_i_*′_(*k*)_ represents the average value of the sequence, *x_i_*_(*k*)_ represents the sequence value, and *x_i_*_(1)_ indicates the average value of the sequence data.

The absolute difference between the main sequence and the subsequence is as shown in Equation (2):

(2) $$\begin{array}{l} \Delta_{i}\left(k\right)=\left|y^{\prime }\left(k\right)-x_{i}^{\prime }\left(k\right)\right|\\ \Delta_{i}\left(k\right)=\{\Delta_{i}\left(1\right),\Delta_{i}\left(2\right),\ldots \ldots \Delta_{i}\left(n\right)\},i=1,2,\ldots \ldots ,m \end{array}$$

where $\Delta_{i}\left(k\right)$ is defined as the absolute difference between the mean values of the principal sequence and the subsequence, $y^{\prime }(k)$ represents the average value of the main sequence, and $x_{i}^{\prime }(k)$ represents the average value of the subsequence.

The maximum value, denoted as *M*, and the minimum value, denoted as *m*, of the absolute difference between the main sequence and the subsequence are defined as shown in Equation (3):

(3) $$M=\underset{i}{\max}\;\underset{k}{\max}\;\Delta_{i}\left(k\right),m=\underset{i}{\min}\;\underset{k}{\min}\;\Delta_{i}\left(k\right)$$

The calculation of the grey relational degree is as shown in Equation (4):

(4) $$\xi_{i}\left(k\right)=\frac{m+\rho M}{\Delta_{i}\left(k\right)+\rho M},k=1,2,\ldots \ldots ,n;i=1,2,\ldots \ldots m$$

where $\xi_{i}\left(k\right)$ represents the grey relational coefficient and ρ is the discrimination coefficient, where ρ = 0.5.

The average value of the grey relational coefficients of each comparison sequence is taken to obtain its relational degree with the reference sequence, as shown in Equation (5):

(5) $$\xi_{i}=\frac{1}{n}\sum_{k=1}^{n}\xi_{i}\left(k\right)$$

where $\xi_{i}$ represents the degree of grey correlation between the main sequence and the subsequence.

The correlation coefficient matrix *ξ*_1_ between the compressive performance of the DRC and the porosity, transition pores, capillary pores, and T_2_ spectrum area is calculated as shown in Equation (6):

(6) $$\xi_{1}=\left[\begin{array}{cccc} 0.958459324 & 0.977772568 & 0.953279575 & 0.333333333\\ 1 & 0.973534698 & 1 & 1\\ 0.967920042 & 0.992765318 & 0.975866891 & 0.733705696\\ 0.968716873 & 1 & 0.967040995 & 0.948198079 \end{array}\right]$$

where *ξ*_1_ represents the grey relational degree between the main sequence and the subsequence.

The grey relational degree between the compressive performance of the DRC and the porosity, transition pores, capillary pores, and T_2_ spectrum area is as shown in Equation (7):

(7) $$\varsigma_{1}=\left[\begin{array}{cccc} 0.8057112 & 0.993383675 & 0.917564487 & 0.970988987 \end{array}\right]$$

The correlation coefficient matrix *ξ*_2_ between the chloride ion penetration resistance of the concrete based on the DRC and the porosity, transition pores, capillary pores, and T_2_ spectrum area is calculated as shown in Equation (8):

(8) $$\xi_{2}=\left[\begin{array}{cccc} 0.966409396 & 0.975777756 & 1 & 0.333333333\\ 0.989418353 & 0.98542731 & 0.981202948 & 1\\ 1 & 0.984149856 & 0.959124994 & 0.910992068\\ 0.977352685 & 1 & 0.986577952 & 0.966236888 \end{array}\right]$$

The degree of grey correlation between the chloride ion penetration resistance of the concrete based on the DRC and the porosity, transition pores, capillary pores, and T_2_ spectrum area is as shown in Equation (9):

(9) $$\varsigma_{2}=\left[\begin{array}{cccc} 0.818880121 & 0.989012153 & 0.96356673 & 0.982541881 \end{array}\right]$$

**Figure 17:**
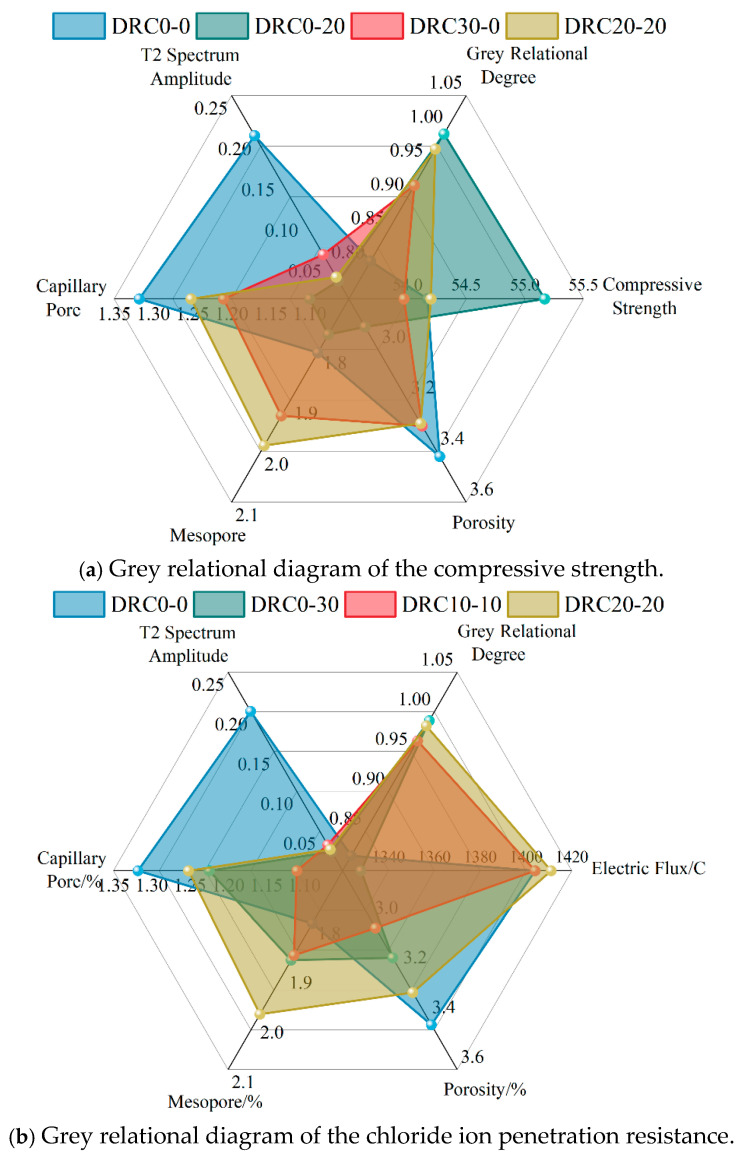
Grey relational degree.

The correlation coefficient value reflects the degree of influence of each index on the chloride ion penetration resistance of the DRC. The range of *ξ* is (0, 1), and the larger the *ξ* value is, the more significant the control effect of each index on the mechanical properties and chloride ion penetration resistance of the concrete [54]. The radar chart of the degree of correlation between the compressive performance and the chloride ion penetration resistance of the DRC is shown in Figure 17. As shown in Figure 17, the control effect of each index on the compressive strength of DRC0-20 is the most significant, and the control effect on the chloride ion penetration resistance of DRC0-30 is the most significant. To obtain better mechanical properties and chloride ion penetration resistance, 20% RA and DS should be added simultaneously.

### 4.2. Economic Benefit Analysis

To assess the economic feasibility of recycled desert sand concrete, the material cost of each mix proportion scheme was calculated on the basis of the local building material market price. The material cost of each mix proportion was calculated according to Equation (10):

(10) $$C=\sum_{i=1}^{n}\left(m_{i}\cdot p_{i}\right)$$

where *C* represents the material cost, *m_i_* is the amount of the *i*-th material, and *p_i_* is the unit price of the *i*-th material.

Research shows that for every 1 ton of recycled aggregate used to replace natural aggregate, CO_2_ emissions can be reduced by 0.015 tons [55], and for every 1 ton of desert sand used to replace river sand, CO_2_ emissions can be reduced by approximately 0.02 tons [56]. Therefore, the carbon emission reduction can be calculated according to Equation (11):

(11) $$M_{CO_{2}}=M_{DS}\times F_{DS}+M_{RA}\times F_{RA}$$

In the formula, *M_DS_* represents the amount of desert sand used, *F_DS_* is the carbon emission reduction factor for desert sand, *M_RA_* represents the amount of recycled aggregate used, and *F_RA_* is the carbon emission reduction factor for RA.

Carbon benefits can be calculated by Equation (12):

(12) $$E_{c}=(M_{DS}\times F_{DS}+M_{RA}\times F_{RA})\times P_{C}$$

In the formula, *E_c_* represents carbon benefits and *P_c_* represents the carbon trading price.

The calculated material costs for each mix ratio are shown in Table 5. The cost of the DS mainly consists of mining and transportation expenses. In this study, the cost of DS is uniformly estimated to be 30 yuan per ton. The cost of the RA is estimated at 60% of the price of natural aggregates. As shown in Table 5, with an increasing proportion of desert sand and recycled aggregates, the material cost of the concrete significantly decreases. When the proportion of recycled aggregates is 30%, the cost savings rate reaches 3.51%. Under the same proportion of recycled aggregates, using 30% DS to replace river sand can further reduce the cost by 1.56%.

### 4.3. Sustainability Analysis

To further clarify the comprehensive technical and economic performance of recycled desert sand concrete, this study conducted cost-effectiveness analyses on compressive strength and chloride ion permeability [57]. The cost-effectiveness index was calculated according to Equations (13) and (14):

(13) $$P_{f,i}=\frac{R_{f,i}}{C_{i}}\times 1000$$

(14) $$P_{d,i}=\frac{R_{d,i}}{C_{i}}\times 1000$$

where *P_f_*_,*i*_ and *P_d_*_,*i*_ are the strength cost-performance index and the chloride ion penetration resistance cost-performance index of the *i*-th mix proportion, respectively; *R_f_*_,*i*_ and *R_d_*_,*i*_ are the grey relational degrees of compressive strength and chloride ion penetration resistance, respectively; and *C_i_* is the material cost. The higher the cost-performance index is, the better the technical performance that can be obtained per unit cost.

**Figure 18:**
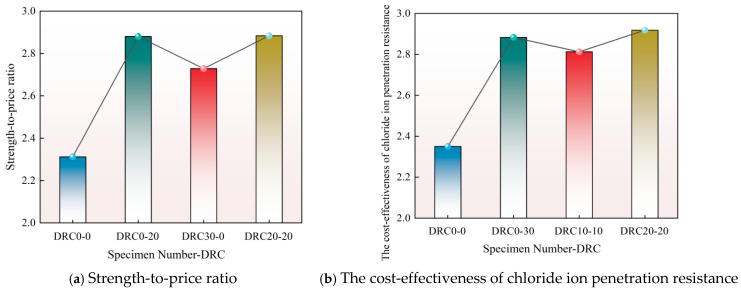
Technical and economic evaluation results.

The calculated strength cost-performance and the chloride ion penetration resistance cost-performance of each mix proportion are shown in Table 6 and Table 7, and Figure 18.

On the basis of the technical and economic evaluation results in Table 6 and Table 7, and Figure 18, DRC20-20 was confirmed to be the optimal mix proportion in this study in terms of comprehensive performance. The data show that the strength cost-effectiveness and impermeability cost-effectiveness of this mix proportion both rank first, with values 24.73% and 24.18%, respectively, higher than those of the benchmark group DRC0-0. This not only validates its excellent performance in the previous grey relational analysis but also highlights its significant cost control advantage.

An in-depth analysis revealed that DRC20-20 can achieve the best balance between performance and cost because, on the one hand, 20% desert sand and 20% recycled aggregate are jointly used, effectively replacing high-priced raw materials and reducing the base material cost; on the other hand, this dosage does not damage the density of the concrete structure, which is consistent with the conclusions obtained from SEM. When the substitution rates of DS and RA are both 20%, the matrix structure is dense, and a large amount of C-S-H gel is generated, which significantly increases the compressive strength and results in high mechanical strength and durability. In contrast, although DRC30-30 has a lower cost, the performance decline leads to a loss of benefits that outweighs the cost savings. Therefore, DRC20-20 represents the optimal solution in terms of the comprehensive technical and economic performance of this material system, providing an ideal material selection basis for balancing performance requirements and budget constraints in practical engineering.

## 5. Conclusions

In this paper, the influence of different rates of DS and RA substitution on the macroscopic performance and microstructure of DRC was systematically studied through tests such as compressive strength, chloride ion permeability, XRD, SEM, and NMR. The grey relational analysis method was adopted to reveal the degree of correlation between pore parameters and mechanical and impermeability performance, clarify the synergistic action mechanism of DS and RA, and determine the optimal mix proportion with the best comprehensive performance through technical and economic analysis. The main conclusions are as follows:

(1) When DS and RA are added together, the 3 d and 7 d compressive strengths of the DRC are higher than those of the control group. The 28 d compressive strength first decreases, then increases, and then decreases with increasing substitution rate. The strength is optimal (reaching 54.20 MPa) when the substitution rates of DS and RA are both 20%. The 6 h chloride ion permeability increases slowly with increasing substitution rate. When the substitution rates of both are 10%, the permeability is the same as that of the control group, and the impermeability performance is the best. If the substitution rate continues to increase, the interface defects of RA will become dominant, and the chloride ion permeability will continue to decrease.

(2) Microscopic pore and phase evolution laws. The hydration products of DRC are mainly C-S-H gel, Ca(OH)_2_, calcite, and ettringite. With the increasing addition of DS and RA, the total porosity decreases, and the pore size distribution is optimized. DS has a microfiller effect, refining the pores and reducing the connectivity between them. Adding a small amount of RA can optimize the pore structure, whereas adding a high amount tends to cause interface defects and microcracks. When the rates of DS and RA addition are both 30%, the porosity is the lowest, and the peak number of small pores is 13.61% lower than that of the control group. The reduction in large pores is the most significant.

(3) The macroscopic performance of the DRC is jointly determined by the microscopic pore structure and interface characteristics. The higher the proportion of small pores, the fewer large pores and through cracks, the denser the matrix, and the better the compressive strength and impermeability. When both DS and RA are 20% of the mixture, the filling effect and skeleton effect are optimally coordinated, with sufficient hydration products, few cracks, and a reasonable pore size distribution, resulting in a simultaneous improvement in the mechanical properties and chloride ion penetration resistance.

(4) Grey relational analysis indicates that pore parameters such as total porosity and transitional pores significantly affect the compressive strength and chloride ion penetration resistance of the DRC. The pore characteristics directly determine the compactness of the matrix and the macroscopic performance. A technical economic and benefit analysis revealed that the cost of DRC decreases significantly with increasing DS and RA dosages and that there is a carbon emission reduction benefit. DRC20-20 has the best strength and impermeability cost performance, which are 24.73% and 24.18% greater than those of the control group, respectively. This is the optimal ratio with balanced technical, economic, and environmental benefits, providing a direct reference for engineering applications.

## Figures and Tables

**Table 1:** Chemical composition of OPC (wt%).

Materials	SiO_2_	Al_2_O_3_	CaO	Fe_2_O_3_	MgO	K_2_O	SO_3_
OPC	25.4	7.1	55.6	4.9	1.3	1.4	3.3

**Table 2:** Physical properties of the coarse aggregates.

Coarse Aggregate	Continuous Gradation/mm	Dry Density/g·cm^−3^	Water Absorption/%
Crushed stone	5–25	2.65	0.45
Recycled aggregate	5–25	2.26	3.2

**Table 3:** Mix Design of Concrete with Desert Sand and Recycled Coarse Aggregate.

Specimen NO.	Consumption of Test Materials/kg·m^−3^						
	OPC	Water	River Sand	Desert Sand	Crushed Stone	Recycled Aggregate	Water Reducer
DRC0-0	420	168	580	0	1320	0	6.3
DRC10-0	420	168	580	0	1188	132	6.3
DRC20-0	420	168	580	0	1056	264	6.3
DRC30-0	420	168	580	0	924	396	6.3
DRC0-10	420	168	522	58	1320	0	6.3
DRC0-20	420	168	464	116	1320	0	6.3
DRC0-30	420	168	406	174	1320	0	6.3
DRC10-10	420	168	522	58	1188	132	6.3
DRC20-20	420	168	464	116	1056	264	6.3
DRC30-30	420	168	406	174	924	396	6.3

Note: In the concrete with recycled coarse aggregate from desert sand (DRC) 0–20, “0” indicates a 0% replacement rate of recycled coarse aggregate, and “20” indicates a 20% replacement rate of desert sand; the numbering of other groups follows this pattern.

**Table 4:** Evaluation of Concrete Permeability Performance.

Permeability	High	Moderate	Low	Very Low	Negligible
QS/C	QS ≥ 4000	2000 ≤ QS < 4000	1000 ≤ QS < 2000	100 ≤ QS < 1000	QS < 100

**Table 5:** Material Costs of Different Mix Proportions.

Specimen NO.	Desert Sand /kg·m^−3^	Recycled Aggregate/kg·m^−3^	Carbon Revenue/Yuan·m^−3^	Material Cost/Yuan·m^−3^	Cost Savings Rate/%
DRC0-0	0	0	0	348.49	0
DRC10-0	0	132	0.12	344.41	1.17
DRC20-0	0	264	0.24	340.33	2.34
DRC30-0	0	396	0.36	336.25	3.51
DRC0-10	58	0	0.07	346.68	0.52
DRC0-20	116	0	0.14	344.87	1.04
DRC0-30	174	0	0.21	343.06	1.56
DRC10-10	58	132	0.19	342.60	1.69
DRC20-20	116	264	0.38	336.71	3.38
DRC30-30	174	396	0.57	330.83	5.07

**Table 6:** Strength-to-price ratio.

Specimen NO.	Compressive Strength Grey Relational Degree	Material Cost/Yuan·m^−3^	Strength-to-Price Ratio
DRC0-0	0.8057	348.49	2.3120
DRC0-20	0.9934	344.87	2.8805
DRC30-0	0.9176	336.25	2.7289
DRC20-20	0.9710	336.71	2.8838

**Table 7:** Cost-effectiveness of resistance to chloride ion penetration.

Specimen NO.	The Grey Correlation Degree of Anti-Chloride Ion Penetration	Material Cost/Yuan·m^−3^	The Cost-Effectiveness of Resistance to Chloride Ion Penetration
DRC0-0	0.8189	348.49	2.3498
DRC0-30	0.9890	343.06	2.8830
DRC10-10	0.9636	342.60	2.8126
DRC20-20	0.9825	336.71	2.9181

## Data Availability

The original contributions presented in this study are included in the article. Further inquiries can be directed to the corresponding author.

## References

[1] Mahdevari S., Abadi A.F.A. (2023). A model based on the evolutionary game theory for implementing green mining principles in riverine sand and gravel resources. J. Clean. Prod..

[2] Liang L., Tong J., Shi Y., Fan X., Xu C. (2025). Experimental study on the influence of particle size and type on single particle breakage of construction waste. Case Stud. Constr. Mater..

[3] Zhang K., Yan G., Liu B., Zhao Y., Liu Y., Yang B. (2025). Aeolian sand concrete for sustainable engineering: Synergistic resistance to freeze-thaw cycles and aeolian sand-gravel flows erosion. Constr. Build. Mater..

[4] Blasenbauer D., Fellner J., Skutan S., Lederer J. (2026). Dry-wet mechanical treatment to produce high-quality recycling aggregate and cement raw material from mixed construction and demolition waste—A material flow analysis case study from Austria. Clean. Waste Syst..

[5] Gong L., Li Z., Ran B., Xu T., Bu Y., Zhao X. (2025). Research on frost resistance of desert sand and sandy sand concrete and lifetime prediction. Results Eng..

[6] Wang Y., Zhu C., Zhang R., Liu H., Liu C., Zhu W., Yu W. (2026). Recycled mixed aggregate concrete: Quadruple interfacial structure system and its influence mechanisms on mechanical properties. J. Build. Eng..

[7] Chen Z., Chen L., Zhao G., Ding S., Huang W., Zhu J., Yang K., Zhu T., Wei F. (2026). Sustainable utilization of recycled aggregates in repairing concrete: Durability behavior under complicated sulfate-magnesium attack. J. Build. Eng..

[8] Al-Jaboury M.F., Dhaheer M.A., Dhahir M.K. (2026). Development of sustainable structural lightweight concrete with fine recycled aggregates. Results Eng..

[9] Yildirim S.T., Meyer C., Herfellner S. (2015). Effects of internal curing on the strength, drying shrinkage and freeze–thaw resistance of concrete containing recycled concrete aggregates. Constr. Build. Mater..

[10] Gao Y., Zhang S., Wang S., Jiang H., Sui H., Zhao L. (2026). Aggregate particle size distribution impact on the microstructure characteristics in the interface transition zone of recycled concrete. Constr. Build. Mater..

[11] Mahi M.A., Zentar R., Becquart F., Sadok A., Gaudron T. (2026). Mechanical, microstructural and environmental performance of 100% recycled-aggregate concrete with low-carbon binders. Results Eng..

[12] Gimenes M., Manzoli O.L. (2026). A novel mesh-fragmentation-based mesoscale approach for modeling compressive fracture in concrete with application to recycled aggregate concrete. Eng. Fract. Mech..

[13] Zheng R., Zhang F., Wang D., Wang M., Wang J. (2025). Basic mechanical properties of geopolymer recycled concrete with iron ore tailings sand. J. Build. Eng..

[14] Yang T., Gong L., Jin C., Qin J., Dang D., Cui X. (2025). Study on the pore characteristics and ITZ properties of recycled aggregate concrete by desert sand subjecting to salt freeze-thaw environments. J. Build. Eng..

[15] Shah M.C., Gupta K.K., Nainwal A., Negi A., Kumar V. (2021). Investigation of mechanical properties of concrete with natural aggregates partially replaced by recycled coarse aggregate (RCA). Mater. Today Proc..

[16] Pico-Molineros A., Sanchez-Rojas E., Bompa D.V., García-Troncoso N. (2026). Incorporation of recycled concrete and mixed recycled aggregate as coarse aggregate replacements in structural grade concrete. Constr. Build. Mater..

[17] Wu E.R., Ma X.K., Fang C.L., Li N., Jia L., Jiang P., Wang W. (2025). Strength performance and microscopic mechanism of cement mortar incorporating fine recycled concrete aggregate and natural sand. J. Build. Eng..

[18] Wan C., Hou P.F., Zhou L., Golewski G.L., Zheng Y.X., Zhang T.H. (2026). The fracture performance of modified recycled concrete: Influence of recycled aggregate and recycled powder. Eng. Fract. Mech..

[19] Zhang Y.D., Huang Y.H., Jiang Z.W., Li Y.Z., Gao Y.Z. (2025). Study on rheological properties and mechanical properties of double-mixed recycled coarse and fine aggregate concrete. AIP Adv..

[20] Zhang M., Zhu X., Shi J., Liu B., He Z., Liang C. (2022). Utilization of desert sand in the production of sustainable cement-based materials: A critical review. Constr. Build. Mater..

[21] Wang D., Che J., Liu C., Liu H. (2024). Design of Mixture Proportion of Engineered Cementitious Composites Based on Desert Sand. KSCE J. Civ. Eng..

[22] Akhtar M.N., Bani-Hani K.A., Malkawi D.A., Albatayneh O. (2024). Suitability of sustainable sand for concrete manufacturing—A complete review of recycled and desert sand substitution. Results Eng..

[23] Akhtar M.N., Albatayneh O., Bani-Hani K.A., Malkawi A.I.H. (2024). Performance of modified desert sand concrete: An experimental case study. Case Stud. Constr. Mater..

[24] Hamada H.M., Abed F., Al-Sadoon Z.A., Elnassar Z., Hassan A. (2023). The use of treated desert sand in sustainable concrete: A mechanical and microstructure study. J. Build. Eng..

[25] Ji Y., Qasem M.G.S., Xu T., Mohammed A.O.Y. (2024). Mechanical properties investigation on recycled rubber desert sand concrete. J. CO_2_ Util..

[26] Liu H., Li L., Tao R., Che J., Zhu L., Sun S., Doh S.I. (2022). Study on the mechanical properties and pore structure of desert sand concrete (DSC) after high temperature. Phys. Chem. Earth Parts A/B/C.

[27] Kazmi S.M.S., Munir M.J., Wu Y.-F. (2025). Durability enhancement of compression-cast desert sand concrete in sulfate-rich environments. J. Build. Eng..

[28] Xu W., Liu H., Qin D., Doh S.I. (2025). Study on the mechanical properties of desert sand concrete under dry-wet cycles with sulfate erosion. Phys. Chem. Earth Parts A/B/C.

[29] Zhang R., Li Z., Ji F., Li Y., Li G., Zhou Y., Zhang H. (2025). Performance study and life prediction of desert sand concrete under chloride salt erosion and freeze-thaw cycle. J. Build. Eng..

[30] Liang C., Bao J., Gu F., Lu J., Ma Z., Hou S., Duan Z. (2025). Determining the importance of recycled aggregate characteristics affecting the elastic modulus of concrete by modeled recycled aggregate concrete: Experiment and numerical simulation. Cem. Concr. Compos..

[31] Chen J., Jia Y., Li Y., Wang Z., Hu Y., Li L. (2026). Research on the evolution mechanism of freeze-thaw damage in hydraulic concrete under different curing humidity conditions based on NMR. Constr. Build. Mater..

[32] Wang D., Zhang H., Chen P., Ju Y., Guo P. (2025). Study on freeze-thaw resistance and pore structure deterioration of fly ash reactive powder concrete based on low-field NMR relaxation. Case Stud. Constr. Mater..

[33] Belebchouche C., Hammoudi A., Moussaceb K., Laouissi A., Hani M., Sahraoui M., Belaadi A., Chetbani Y., Alshaikh I.M., Ghernaout D. (2026). Modeling of recycled coarse aggregates concrete characteristics via hybrid improved Grey Wolf Optimizer deep neural network and multi-objective Grey Wolf Optimization. Results Eng..

[34] Ren Z., Dong W., Dong X. (2025). Analysis of fractal and erosion characteristics of aeolian sand concrete pore structure under capillary absorption. J. Build. Eng..

[35] Zhu W., Li Y., Zheng X., Hao E., Zhang D., Wang Z. (2025). Prediction of compressive strength and characteristics analysis of semi-flexible pavement desert sand grouting material based upon hybrid-BP neural network. Case Stud. Constr. Mater..

[36] (2019). Specification for Mix Proportion Design of Ordinary Concrete.

[37] (2019). Standard for Test Methods of Concrete Physical and Mechanical Properties.

[38] (2024). Standard for Test Methods of Long-Term Performance and Durability of Ordinary Concrete.

[39] Sharma R., Senthil K. (2023). An investigation on mechanical and microstructural properties of hybrid fiber reinforced concrete with manufactured sand and recycled coarse aggregate. J. Build. Eng..

[40] Sun H., Luo L., Li X., Yuan H. (2024). The treated recycled aggregates effects on workability, mechanical properties and microstructure of ultra-high performance concrete Co-reinforced with nano-silica and steel fibers. J. Build. Eng..

[41] (2012). Standard Test Method for Electrical Indication of Concrete’s Ability to Resist Chloride Ion Penetration.

[42] Kouřil M., Saksa J., Hybášek V., Sedlářová I., Němeček J., Kohoutková M., Němeček J. (2025). Corrosion Response of Steel to Penetration of Chlorides in DC-Treated Hardened Portland Cement Mortar. Materials.

[43] Dong W., Sun A., Wang X. (2024). NMR-based analysis of fractal characteristics of the pore structure of fully aeolian sand concrete under carbonation-dry-wet cycles. Mater. Today Commun..

[44] Yan J., Feng L., Liu S., Zhang Z., Yang B., Xie J., Wang J., Zhao T., Xing Z. (2026). Effect of glass microfibers on mechanical properties and chloride ion permeability resistance of recycled aggregate concrete. Constr. Build. Mater..

[45] Jaya N.A., Yun-Ming L., Cheng-Yong H., Abdullah M.M.A.B., Hussin K. (2020). Correlation between pore structure, compressive strength and thermal conductivity of porous metakaolin geopolymer. Constr. Build. Mater..

[46] Shen Y., Ueda T., Wang Y. (2025). Multiscale pore structure characterization and permeability analysis based on concrete NMR-CT registration method. Constr. Build. Mater..

[47] Liang N., Geng S., Mao J., Liu X., Zhou X. (2024). Investigation on cracking resistance mechanism of basalt-polypropylene fiber reinforced concrete based on SEM test. Constr. Build. Mater..

[48] Dai J., Yin S. (2025). Mechanical properties of hybrid fiber reinforced coral aggregate seawater concrete based on scanning electron microscopy (SEM). J. Build. Eng..

[49] Luo F.J., He L., Pan Z., Duan W.H., Zhao X.L., Collins F. (2013). Effect of very fine particles on workability and strength of concrete made with dune sand. Constr. Build. Mater..

[50] Chuah S., Duan W.H., Pan Z., Hunter E., Korayem A.H., Zhao X.L., Collins F., Sanjayan J.G. (2016). The properties of fly ash based geopolymer mortars made with dune sand. Mater. Des..

[51] Zhu W., Liu R., Zheng X., Cao Y., Zhang D., Pei Y., Chang D. (2025). Durability evaluation and life prediction of desert sand grouting material for semi—Flexible pavements under sulfate dry—Wet cycles based on entropy weight method and grey theory. Case Stud. Constr. Mater..

[52] Wang K., Wang W., Guo Y., Liu Y., Duan P., Shi W., Liu Y. (2024). Grey modeling study on mechanical properties and pore structure of concrete with different basalt fiber contents based on NMR. J. Build. Eng..

[53] Cheng X., Tian W., Gao J., Guo J., Wang X. (2022). Grey entropy analysis of strength and void structure of carbon nanotubes concrete under the coupling of sulfate attack and freeze-thaw cycles. Constr. Build. Mater..

[54] Zhou M., Dong W. (2023). Grey correlation analysis of macro- and micro-scale properties of aeolian sand concrete under the salt freezing effect. Structures.

[55] Akbas M., Akin F.D. (2025). A comparative weighting analysis using AHP and CRITIC for recycled pavement material selection: A case study from Istanbul. Results Eng..

[56] Hamada H.M., Abed F., Tracy K., Tayeh B.A. (2025). Utilizing Raw Desert Sand as a Sustainable Fine Aggregate: Impact on Concrete Performance and Environmental Benefits. Int. J. Concr. Struct. Mater..

[57] Bavithra K., Mohana R. (2025). Sustainable development of durable and novel nano GGBS impregnated eco-friendly green concrete using micro structural characterization and techno-economic sustainability analysis. Constr. Build. Mater..

